# Long noncoding RNA MRCCAT1 promotes metastasis of clear cell renal cell carcinoma via inhibiting NPR3 and activating p38-MAPK signaling

**DOI:** 10.1186/s12943-017-0681-0

**Published:** 2017-06-28

**Authors:** Jia-Kuan Li, Cheng Chen, Jia-Yi Liu, Jia-Zi Shi, Shu-Peng Liu, Bing Liu, Deng-Shuang Wu, Zi-Yu Fang, Yi Bao, Ming-Ming Jiang, Ji-Hang Yuan, Le Qu, Lin-Hui Wang

**Affiliations:** 1 Department of Urology, Changzheng Hospital, Second Military Medical University, 415 Fengyang Road, Shanghai, 200003 China; 2 Department of Urology, The 517th Hospital of People’s Liberation Army, Shanxi, 036301 China; 3 Department of Medical Oncology, Jinling Hospital, Nanjing University Clinical School of Medicine, Nanjing, 210002 China; 4 Central laboratory, Changhai Hospital, Second Military Medical University, Shanghai, 200433 China; 5 Department of Urology, Changhai Hospital, Second Military Medical University, Shanghai, 200433 China; 6 Clinical laboratory, Eastern Hepatobiliary Surgery Hospital, Second Military Medical University, Shanghai, 200433 China; 7 Department of Medical Genetics, Second Military Medical University, Shanghai, 200433 China; 8 Department of Urology, Jinling Hospital, Nanjing University Clinical School of Medicine, Nanjing, 210002 China

**Keywords:** Clear cell renal cell carcinoma, Long noncoding RNA, Metastasis, MAPK signaling

## Abstract

**Background:**

Recent evidences showed that long noncoding RNAs (lncRNAs) are frequently dysregulated and play important roles in various cancers. Clear cell renal cell carcinoma (ccRCC) is one of the leading cause of cancer-related death, largely due to the metastasis of ccRCC. However, the clinical significances and roles of lncRNAs in metastatic ccRCC are still unknown.

**Methods:**

lncRNA expression microarray analysis was performed to search the dysregulated lncRNA in metastatic ccRCC. quantitative real-time PCR was performed to measure the expression of lncRNAs in human ccRCC samples. Gain-of-function and loss-of-function experiments were performed to investigate the biological roles of lncRNAs on ccRCC cell proliferation, migration, invasion and in vivo metastasis. RNA pull-down, RNA immunoprecipitation, chromatin immunoprecipitation, and western blot were performed to explore the molecular mechanisms underlying the functions of lncRNAs.

**Results:**

The microarray analysis identified a novel lncRNA termed metastatic renal cell carcinoma-associated transcript 1 (MRCCAT1), which is highly expressed in metastatic ccRCC tissues and associated with the metastatic properties of ccRCC. Multivariate Cox regression analysis revealed that MRCCAT1 is an independent prognostic factor for ccRCC patients. Overexpression of MRCCAT1 promotes ccRCC cells proliferation, migration, and invasion. Depletion of MRCCAT1 inhibites ccRCC cells proliferation, migration, and invasion in vitro, and ccRCC metastasis in vivo. Mechanistically, MRCCAT1 represses *NPR3* transcription by recruiting PRC2 to *NPR3* promoter, and subsequently activates p38-MAPK signaling pathway.

**Conclusions:**

MRCCAT1 is a critical lncRNA that promotes ccRCC metastasis via inhibiting NPR3 and activating p38-MAPK signaling. Our results imply that MRCCAT1 could serve as a prognostic biomarker and therapeutic target for ccRCC.

**Electronic supplementary material:**

The online version of this article (doi:10.1186/s12943-017-0681-0) contains supplementary material, which is available to authorized users.

## Background

Renal cell carcinoma (RCC) is the second leading cause of death associated with urological malignant neoplasms. Approximately 80-90% of RCCs are clear cell renal cell carcinoma (ccRCC), with a characteristic of high metastasis and relapse rate compared with other RCC subtypes [1]. For early stage RCC, partial nephrectomy is generally used as the standard approach to remove localized RCC with a good prognosis [2]. However, the prognosis of patients with metastatic RCC is extremely poor. The 5-year survival rate of metastatic RCC is only 10%, and while that of non-metastatic RCC is estimated to be approximately 55% [3]. At present, there is a lack of effective biomarker for metastasis prediction and drug target for therapeutic intervention. Therefore it is necessary to identify new sensitive, reliable biomarker enabling the prediction of progression and prognosis, and to develop new targeted therapies for metastatic ccRCC.

The rapid development of genomics and transcriptomics highlighted the important role of noncoding RNAs (ncRNAs) in human tumors. Long noncoding RNAs (lncRNAs) are RNA molecules with more than 200 nucleotides in length and no protein production [3]. lncRNAs have been reported to play important roles in diverse cellular processes, such as cell growth, apoptosis, migration, invasion, and autophagy [4–6]. Specific lncRNAs have also been demonstrated to promote the development of human cancers. For example, the lncRNA HOTAIR, which is known to regulate the expression of HOX gene clusters, is highly expressed in breast cancer samples, and its elevated expression is correlated with metastasis and death [7]. The lncRNA TUG1 has potential roles as a biomarker and a therapeutic target in bladder urothelial carcinoma [8]. The lncRNA SPRY4-IT1, which is increased in esophageal squamous cell carcinoma tissues, is significantly associated with the clinical pathological stages and the overall survival rate of the disease [9].

Although multiple lncRNAs have been reported to modulate tumor development and metastases [10], the functional roles of lncRNAs in ccRCC remain largely unknown. In this study, we report the identification of a novel lncRNA ENST00000505584 which is upregulated in metastatic ccRCC and associated with poor prognosis of ccRCC patients. Hereafter, we termed this lncRNA as metastatic renal cell carcinoma-associated transcript 1 (MRCCAT1). Functional experiments showed that MRCCAT1 promotes proliferation, migration and invasion of ccRCC cells in vitro and in vivo. Mechanistically, we found that MRCCAT1 negatively regulates C5orf23 (namely NPR3, NPRC or NPR-C) expression. NPR3 is negatively coupled to adenylyl cyclase and MAP kinase pathway (mitogen-activated protein kinase, MAPK) [11–13]. Thus we conjectured that MRCCAT1 may positively participate in the adenylyl cyclase and MAPK signaling pathway by inhibiting NPR3 expression.

## Methods

### Patients and clinical samples

A total of 34 metastatic and 34 non-metastatic ccRCC primary tissues were collected from patients who underwent surgery for ccRCC at Changzheng Hospital, Second Military Medical University (Shanghai, China), including 6 cases of ccRCC tissue samples (3 metastatic tumor samples and 3 non-metastatic tumor samples) used in this study for lncRNA microarray analysis. These tissues were flash frozen in liquid nitrogen immediately after surgery and subsequently stored at −80 °C. All the resected nodules were identified by pathologic examination. No patients received anticancer treatments before surgery in this study. All samples were staged according to the tumor node metastasis (TNM) classification and criteria of World Health Organization (WHO), and tumor grade was assessed in accordance with the WHO criteria. The median fellow-up time of these 68 ccRCC patients is 52 month. Written informed consent was obtained from all patients. The Ethics Committee of Changzheng Hospital, Second Military Medical University approved the use of these tissues in this study.

### Microarray analysis

Microarray analysis for the expression of lncRNAs and mRNAs was performed by Shanghai Gminix Biological Information Company (Shanghai, China), using Affymetrix glue grant human transcriptome array. The accession number for the microarray data reported in this paper is Gene Expression Omnibus database GEO: GSE88948.

### Target gene prediction and lncRNA-mRNA co-expression network

To further elucidate correlations between lncRNAs and mRNAs, potential lncRNA-mRNA co-expression analysis was conducted according to the normalized signal intensity of specific expression genes.

### Plasmid construction and cell transfection

Plasmid pcDNA3.1^+^-MRCCAT1 and pcDNA3.1^+^-vector were purchased from BioBoer Technologies (Wuhan, China). Short hairpin RNA (shRNA) sequences were designed by Hanbio Biotechnology Co. Ltd. (Shanghai, China) to target human MRCCAT1. After annealing, double strands of shRNA were inserted into lentiviral pHBLV-U6-Scramble-Luc-Puro vector (Hanbio, Shanghai, China), named Lv-shMRCCAT1, and negative control was named Lv-shNC. Human full-length cDNA of NPR3 was cloned into expression plasmid pLVX-MCS-3flag-IRES-ZsGreen1-PGK-Puro (Genomeditech, Shanghai, China), named pLVX-NPR3, and the empty pLVX-puro lentiviral expression vector was used as control (pLVX-vector).

Plasmid transfections were performed using Lipofectamine 2000 (Invitrogen, Carlsbad, CA, USA) according to the manufacturer’s protocol. Cells were incubated for 24 h before use in experiments. Stable cell lines were generated by using puromycin. The sequences of primers used for plasmid construction in this study were provided in Additional file 1: Table S1.

### Cell lines and reagents

The human ccRCC cell lines 786-O and Caki-1 were purchased from American Type Culture Collection (ATCC, Rockville, MD, USA) and were cultured in RPMI-1640 medium (Gibco, Thermo Fisher Scientific, Waltham, MA, USA) and McCoy’s 5A medium (Gibco) and supplemented with 10% fetal bovine serum (Gibco).

### RNA extraction and quantitative real-time PCR (qRT-PCR)

The total RNA of the tissue samples was extracted using the Trizol reagent (Invitrogen) according to the manufacturer’s instructions. cDNA was converted from total RNA by using a Reverse Transcription Kit (Takara, Dalian, China) according to the manufacturer’s instructions. Quantitative real-time PCR was performed with SYBR Green (Takara, Dalian, China), and the data collection was performed on the Applied Biosystems® 7500 Real-Time PCR Systems (Thermo Fisher Scientific) according to the manufacturer’s instructions. The primers were synthesized by Biosune (Shanghai, China). The relative expression level of indicated genes was compared with that of β-actin and expression fold changes were calculated using 2^-△△Ct^ methods [14]. Each qRT-PCR reaction was performed in triplicate. Sequences of primers used for qRT-PCR in this study are shown in Additional file 1: Table S2.

### 5′ and 3′ rapid amplification of cDNA ends (RACE)

We performed the 5′-RACE and 3′-RACE analyses to determine the transcriptional initiation and termination sites of MRCCAT1 using a SMARTer™ RACE cDNA Amplification Kit (Clontech, Palo Alto, CA, USA) according to the manufacturer’s instructions. The gene-specific primers used for the PCR of the RACE analysis were 5′-GGTCATTGGAGTCAGTGCTCTC-3′ (5′-RACE) and 5′-CGCTGTATCTTCTCCTCAGGTATG-3′ (3′-RACE).

### Cell proliferation assay

Cell proliferation was assessed using a cell proliferation kit, Cell Counting Kit-8 (CCK-8; Dojindo Molecular Technologies, Inc., Kyushu, Japan), according to the manufacturer’s instructions. Cells were seeded into 96-well culture plates at a density of 2 × 10^3^ cells/well the day before transfection. Approximately 10 μl of CCK8 regent was added to each well after transfection and incubated at 37 °C for 2 h. Cell growth was analyzed at a wave length of 450 nm at 24, 48, 72, and 96 h after transfection using Spectra Max M3 (Molecular Devices). Experiments were performed in triplicate.

### Wound healing assay

ccRCC cells were seeded at 5 × 10^5^ cells/well in 6-well plates and cultured until the plates were confluent. The cell monolayer was scraped in a straight line using a 10 μl pipette tip to create a scratch, washed with PBS twice and the medium was replaced with serum-free medium. Images were captured at 0, 24(18), and 48(36)h following the initial scratch to evaluate cell migration.

### Transwell assay

The invasion of ccRCC cells was assessed based on the number of cells through Matrigel-coated Transwell inserts, as previously described [15]. In brief, 3 × 10^5^ cells were seeded into 24-well plate-sized inserts (a chamber containing a pore size of 8 μm, Corning Life Sciences, NY, USA) with Matrigel (BD Biosciences, San Jose, CA, USA). Cells were plated in medium without serum, with the lower chamber containing the medium plus 10% FBS, and thus serving as a chemo-attractant. After incubation for 24 h, the cells that did not invade through the pores were carefully wiped out with cotton wool. All cells that had migrated from the upper to the lower side of the filter were fixed with 4% paraformaldehyde and stained with 1% crystal violet, and then counted and imaged (magnification, ×100). The assay was conducted three separate times.

### Western blot analysis

Western blot was conducted under standard procedures [16]. Briefly, cells were lysed to obtain proteins using RIPA. Proteins were separated by 8% SDS–PAGE and then transferred to PVDF membrane (Bio-Rad, Hercules, CA, USA). After blocking in 5% nonfat milk, the membranes were incubated with the following primary antibodies: NPR3 (Proteintech-25,248-1-AP), p-ERK, p-JNK, p-p38 MAPK, total ERK1/2, total JNK, total p38 MAPK (Cell Signaling Technology, Boston, MA, USA), and β-actin (Santa cruz-81,178); then the following secondary antibodies were used: rabbit anti-mouse IgG and goat anti-rabbit IgG (SAB, Bethesda, MD, USA). All antibodies were diluted in nonfat dry milk. The immunoreactive protein bands were visualized by ECL Kit (Pierce, Thermo Fisher Scientific, IL, USA). The experiment was performed three separate times.

### RNA immunoprecipitation (RIP)

We performed RNA immunoprecipitation (RIP) experiments using the Magna RIP RNA-Binding Protein Immunoprecipitation Kit (Millipore, Bedford, MA, USA) according to the manufacturer’s instructions. The EZH2 antibody used for RIP was clone AC22 (17-662; Millipore). An aliquot of lysate was removed as an input control. RNA enrichment was determined by qRT-PCR and normalized to the input control.

### RNA pull-down assay

RNA pull-down was performed as described previously [17]. Briefly, biotin-labeled RNAs were transcribed with the Biotin RNA Labeling Mix (Roche Diagnostics, Indianapolis, IN, USA) and T7/SP6 RNA polymerase (Roche), treated with RNase-free DNase I (Roche), and purified with the RNeasy Mini Kit (Qiagen, Inc., Valencia, CA, USA). Cell nuclear proteins were extracted using the Proteo JETTM Cytoplasmic and Nuclear Protein Extraction Kit (Fermentas, St. Leon-Rot, Germany). One milligram of 786-O or Caki-1 cell nuclear extract was then mixed with 50 pmol of biotin-labeled RNAs. 60 μl of washed streptavidin agarose beads (Invitrogen) were added to each binding reaction and further incubated at room temperature for 1 h. Beads were washed briefly five times and boiled in sodium dodecyl sulfate buffer, and the retrieved protein was detected by the standard western blot technique.

### Chromatin immunoprecipitation (ChIP)

Chromatin immunoprecipitation (ChIP) was performed using the EZ ChIP™ Chromatin Immunoprecipitation Kit (Millipore), according to the manufacturer’s instructions. Briefly, cross-linked chromatin was sonicated into 200-1000 bp fragments. The chromatin was immunoprecipitated using anti-EZH2 (Millipore), anti-H3K27me3 (Millipore), anti-RNA Pol II antibodies. Normal mouse immunoglobulin G (IgG) was used as negative control. qRT-PCR was conducted using SYBR Green (Takara) and the gene-specific primers with the sequences 5′-GCCTGCGGGGAGTGGTGGT-3′ (forward) and 5′-CCGAGGCCGGGCTTGTGTT-3′ (reverse).

### In vivo metastatic tumor experiment

Male nude mice (BALB/c Nude; 4 weeks old) were purchased from the Shanghai Institute of Material Medical (Chinese Academy of Science, Shanghai, China) and maintained in a pathogen-free condition in accordance with relevant guidelines and regulations for the care and use of laboratory animals, with the approval of the Institutional Animal Care and Use Committee at Second Military Medical University. To establish a metastatic cancer model in vivo, 786-O cells infecting Lv-shMRCCAT1 and 786-O cells infecting Lv-shNC stably expressing luciferase were elected by puromycin (2 μg/ml). Cells (2 × 10^6^) in 200 μl PBS were injected into the tail vein of mice. Metastatic progression was monitored weekly and quantified using a noninvasive bioluminescence In-Vivo Imaging System (IVIS; Xenogen) 10 min after intraperitoneal injection of 4.0 mg of luciferin (Gold Biotech) in 50 μl of saline, as previously described [18, 19]. Mice were sacrificed at 6 weeks after inoculation, and consecutive sections of the whole lung were subjected to hematoxylin-eosin staining. In addition, lungs with metastatic renal cell carcinomas were measured and used for further analysis, such as Western blot.

### Statistical analysis

Statistical analysis was performed with SPSS Statistics software version 13 (SPSS Inc., USA). Data were presented as the mean ± SD or the mean rank. The repeated measures method was used to analyze the proliferation plot. The Kaplan-Meier method and log-rank tests were used to compare ccRCC patient survival based on dichotomized MRCCAT1 expression. Cox proportional hazards regression analysis was used to analyze the independent factors on the survival prognosis of patients with ccRCC. Differences between groups were analyzed by Student’s t-test or nonparametric Mann-Whitney U test. A value of *P* < 0.05 was considered significant.

## Results

### Identification of MRCCAT1 which is upregulated in metastatic ccRCC tissues

**Fig. 1:**
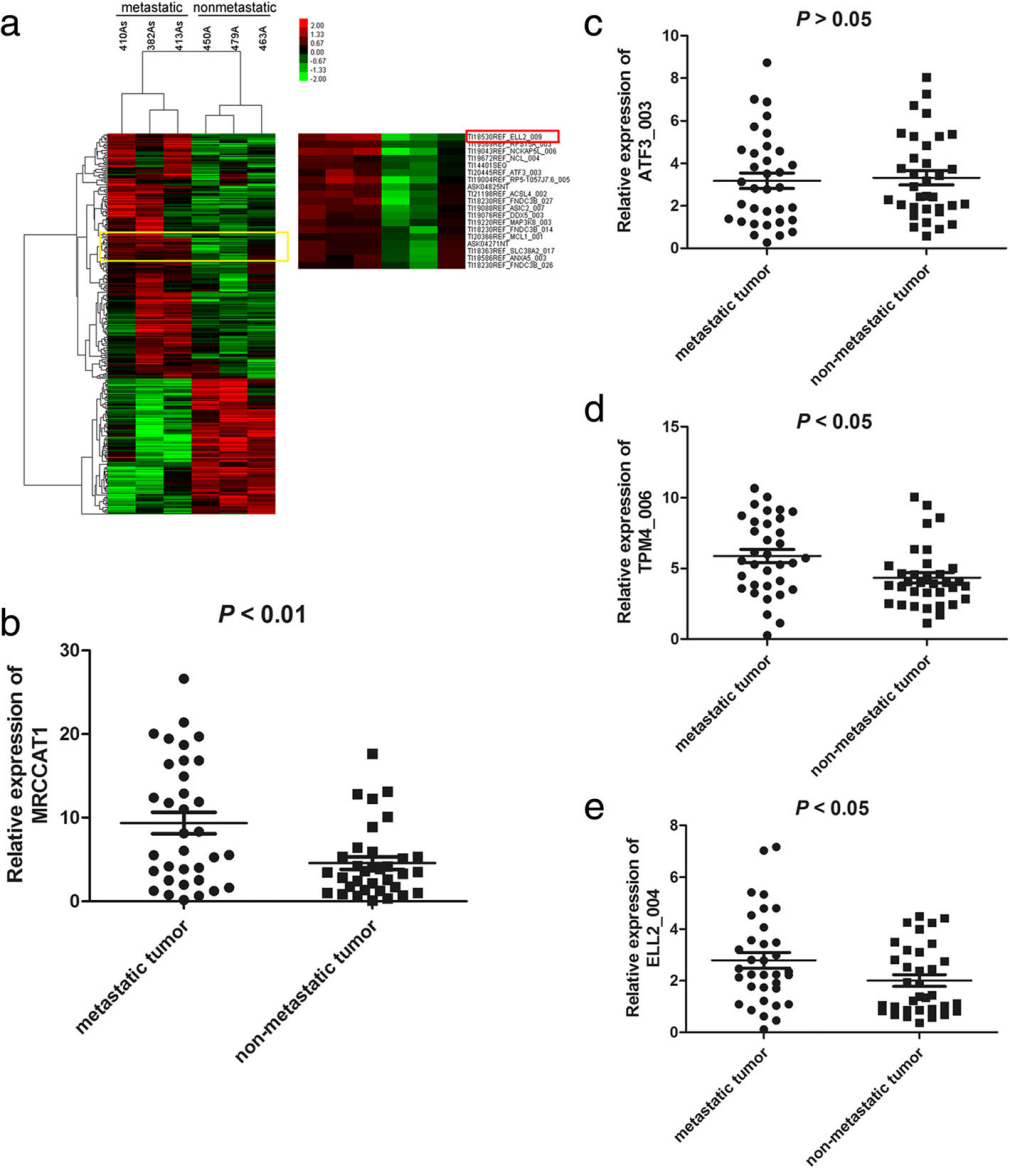
Identification of MRCCAT1 which is upregulated in metastatic ccRCC tissues. **a** lncRNAs microarray data of metastatic ccRCC samples compared with that of non-metastatic ccRCC samples are presented in a heat map. Red represents high expression, and green represents low expression. MRCCAT1 (TI18530REF_ELL2_009) is listed on the right. **b** qRT-PCR analysis of MRCCAT1 between metastatic and non-metastatic ccRCC samples. *P* < 0.01 by Mann-Whitney U test. **c** qRT-PCR analysis of ATF3_003 between metastatic and non-metastatic ccRCC primary samples. *P* > 0.05 by Mann-Whitney U test. **d** qRT-PCR analysis of TPM4_006 between metastatic and non-metastatic ccRCC primary samples. *P* < 0.05 by Mann-Whitney U test. **e** qRT-PCR analysis of ELL2_004 between metastatic and non-metastatic ccRCC primary samples. *P* < 0.05 by Mann-Whitney U test

To identify lncRNAs essential for ccRCC metastasis, 3 metastatic ccRCC tissues and 3 non-metastatic ccRCC tissues were subjected to lncRNA expression microarray. Cluster analysis showed a clear distinction between metastatic ccRCC tissues and non-metastatic ccRCC tissues (Fig. 1a). We identified 206 upregulated lncRNAs and 114 downregulated lncRNAs (*P* value <0.0001, fold change >2). Among the upregulated lncRNAs in metastatic ccRCC, TI18530REF_ELL2_009 (ENST00000505584) is found to be significantly higher in 34 metastatic ccRCC tissues than that in 34 non-metastatic ccRCC tissues (Fig. 1b). Compared with TI20445REF_ATF3_003, TI18717REF_TPM4_006 and TI18530REF_ELL2_004, the expression difference of TI18530REF_ELL2_009 is more significant (Fig. 1b-e). As a result, we named TI18530REF_ELL2_009 as metastatic renal cell carcinoma-associated transcript 1 (MRCCAT1).

### MRCCAT1 upregulation is associated with aggressive clinicopathological traits and serves as a prognostic factor for ccRCC patients

**Fig. 2:**
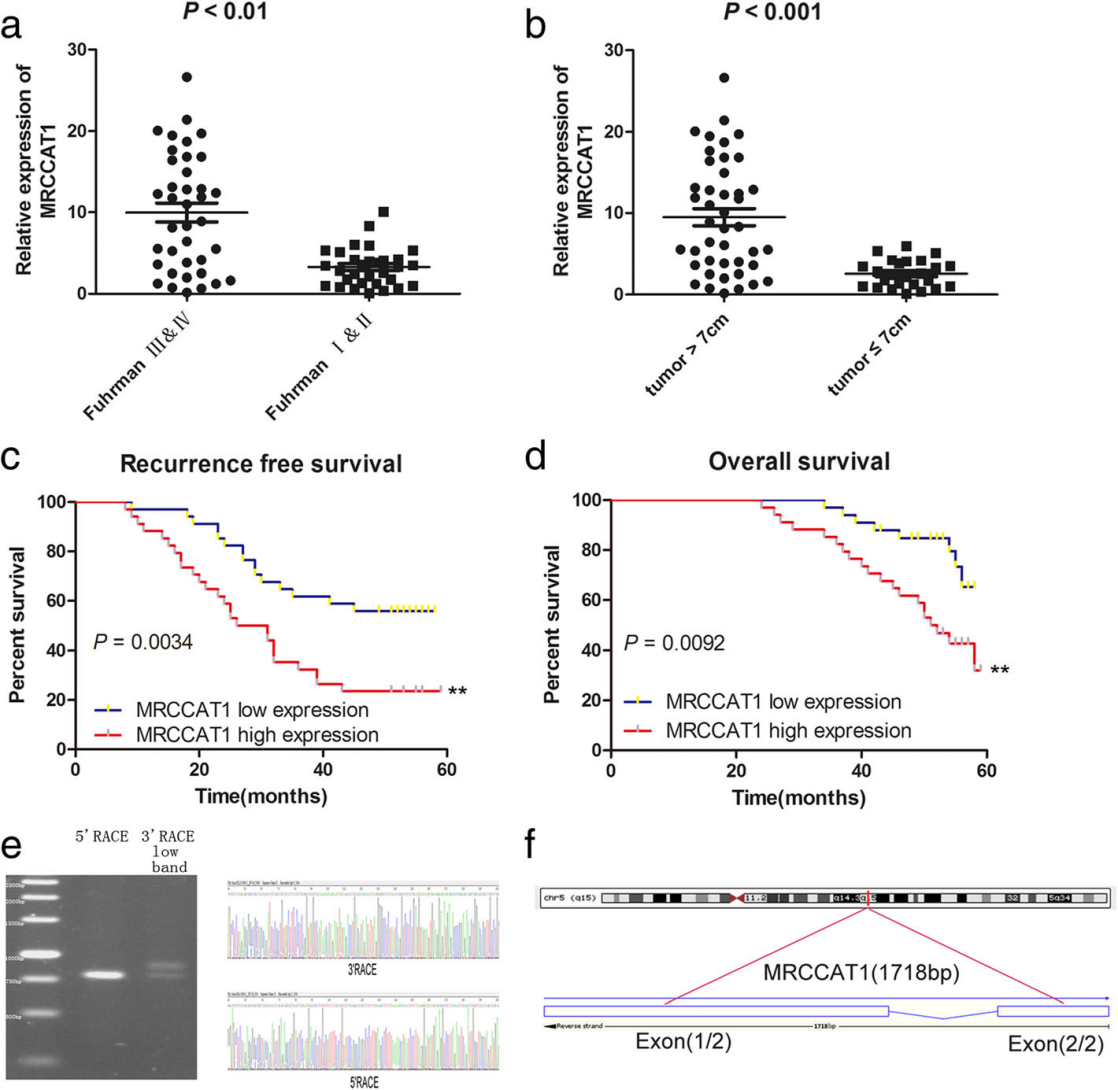
The association between MRCCAT1 and clinicopathological characteristics of ccRCC patients. **a** qRT-PCR analysis of MRCCAT1 between Fuhrman III-IV grade and Fuhrman I-II grade ccRCC samples. *P* < 0.01 by Mann-Whitney U test. **b** qRT-PCR analysis of MRCCAT1 between tumor >7 cm and tumor ≤7 cm ccRCC samples. *P* < 0.01 by Mann-Whitney U test. **c** Patients in the MRCCAT1 high expression group (*n* = 34) had shorter recurrence free survival time than those in the MRCCAT1 low expression group (*n* = 34). *P* = 0.0034 by Log-rank test. **d** Patients in the MRCCAT1 high expression group (*n* = 34) had shorter overall survival time than those in the MRCCAT1 low expression group (*n* = 34). *P* = 0.0092 by Log-rank test. **e** The 5′-RACE and 3′-RACE assays were performed to determine the transcriptional initiation and termination sites of MRCCAT1. Left, representative images of PCR products from the 5′-RACE and 3′-RACE procedure. Right, sequence of the second-round PCR products revealed the boundary between the universal anchor primer and the MRCCAT1 sequences. **f** Schematic annotation of MRCCAT1 genomic locus on chromosome 5: 95,900,228-95,901,132 and composed of 2 exons in humans. Blue rectangles represent exons

Analyses of the correlation between MRCCAT1 expression and clinicopathological traits showed that MRCCAT1 expression is much higher in Fuhrman III and IV grades than Fuhrman I and II grades (*P* = 0.001); and in tumors >7 cm than tumors ≤7 cm (*P* = 0.007) (Fig. 2a and b). There is no significant correlation between MRCCAT1 expression with age (*P* = 0.091) or gender (*P* = 0.397) (Table 1). These data suggest that high MRCCAT1 expression may have an important role in ccRCC progression and metastasis.

**Table 1:** Correlation between clinicopathological features and the expression of MRCCAT1 and NPR3

Clinicopathological features	Patients *n* = 68	MRCCAT1 expression mean rank	*P* value	NPR3 expression mean rank	*P* value
Age (y)			0.091		0.324
< 60	43	31.41		32.70	
≥ 60	25	39.82		37.6	
Gender			0.397		0.978
Male	49	33.23		34.46	
Female	19	37.76		34.41	
Tumor size (cm)			0.007**		0.01*
≤ 7	50	30.6		38.19	
> 7	18	45.33		24.24	
Fuhrman tumor grade			0.001**		0.002**
I-II	41	27.93		40.39	
III-IV	27	44.48		25.56	
Metastasis			0.008**		0.0001***
Yes	34	40.87		25.53	
No	34	28.13		43.47	

*P < 0.05, **P < 0.01, ***P < 0.001

We also compared the survival time following surgery in these 68 cases of ccRCC patients with higher MRCCAT1 expression (above the median value, *n* = 34) with those with lower MRCCAT1 expression (below the median value, *n* = 34). As shown in Fig. 2c and d, cumulative recurrence free survival and overall survival rates were significantly better in patients with lower MRCCAT1 expression than in those with higher MRCCAT1 expression. Additionally, univariate analysis showed that patients with high MRCCAT1 expression, metastases, high Fuhrman grade, and large tumor size were significantly associated with an increased risk of cancer-related death. Multivariate analysis identified high expression level of MRCCAT1 as an independent prognostic factor for ccRCC patients (Table 2). These data indicated that MRCCAT1 expression is a new prognostic factor for ccRCC patients.

**Table 2:** Univariate and multivariate analyses of factors associated with overall survival in ccRCC patients

Variable	Univariate	Multivariate		
		Hazard ratio	95% CI	*P* value
MRCCAT1 expression High vs. Low	0.002	2.306	1.003-2.849	0.019*
NPR3 expression High vs. Low	0.04	0.645	0.309-1.349	0.244
Fuhrman grade III/VI vs. I/II	0.006	0.824	0.364-1.868	0.643
Tumor size >7 cm vs. ≤7 cm	0	1.117	0.326-3.825	0.86
Metastasis Yes vs. No	0.001	4.035	1.055-15.434	0.042*

*P < 0.05

Since actual sequences of lncRNAs generally differ from their predicted sequences [20–22], we determined the transcriptional initiation and termination sites of MRCCAT1 using 5′- and 3′-RACE. As shown in Fig. 2e and Additional file 2: Figure S1a, the full-length of MRCCAT1 is 1718 bp, longer than the predicted sequences. MRCCAT1 includes two exons that are located at Chromosome 5:95,900,228-95,901,132 (Fig. 2f). ORF Finder (National Center for Biotechnology Information) failed to predict a protein of more than 62 amino acids, as shown in Additional file 2: Figure S1b. Consistently, the txCdsPredict score of MRCCAT1 is 245.0, supporting that MRCCAT1 has no protein-coding potential. We also performed a codon substitution frequency analysis using PyhloCSF [23, 24]. MRCCAT1 showed very low codon substitution frequency scores (−0.35), indicating that it is a non-coding RNA (Additional file 2: Figure S1c). Furthermore, CNCI software (The Broad Institute, Cambridge, MA,USA) showed that MRCCAT1 did not contain a valid Kozak sequence and is identified to be an lncRNA, rather than a protein-coding transcript, with a score of −0.19 [25]. Thus, we identified a novel lncRNA highly expressed in metastatic ccRCC.

### MRCCAT1 promotes ccRCC cells migration and invasion in vitro

**Fig. 3:**
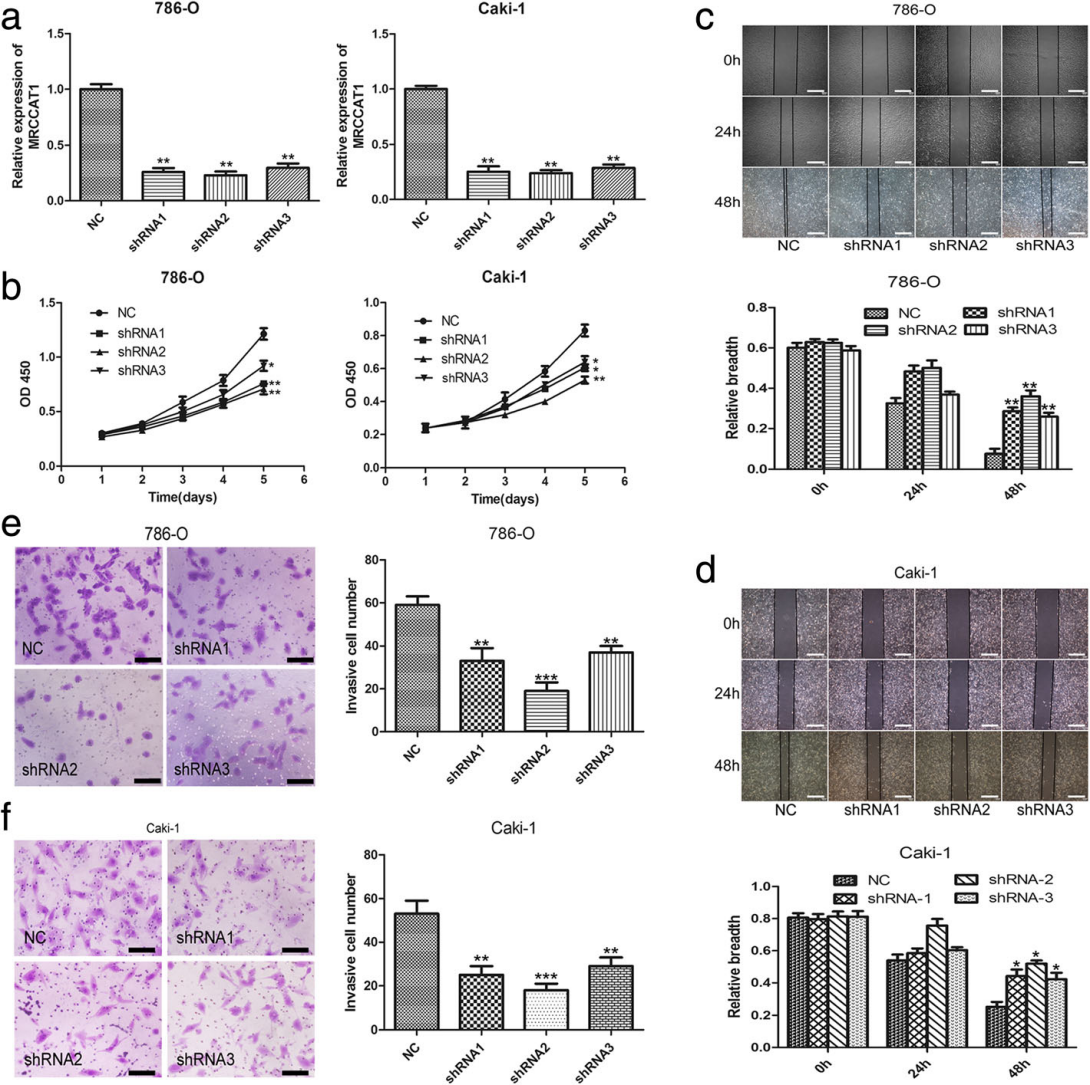
MRCCAT1 knockdown suppresses ccRCC cells proliferation, migration and invasion in vitro. **a** Relative expression of MRCCAT1 in 786-O and Caki-1 cells after transfecting with MRCCAT1 shRNAs compared with negative control (NC). **b** CCK-8 assay of MRCCAT1 knockdown (shRNAs) and NC ccRCC cells at indicated times. **c**, **d** Upper: representative images of the wound-healing assays of MRCCAT1 knockdown with shRNAs or NC in 786-O (**c**) and Caki-1 cells (**d**) photographed at indicated times after scratching. Scale bar = 800 μm. Lower: relative breath after migration was measured and calculated among the groups. **e**, **f** Left: Transwell assays were performed to evaluate cell invasion following MRCCAT1 knockdown or NC in 786-O cells (**e**) and Caki-1 cells (**f**). Scale bar = 200 μm. Right: the statistical graph indicates the number of cells averaged from 8 random high power fields. The results are presented as the mean ± SD from three independent experiments; *P < 0.05, **P < 0.01 by Student’s t-test

To investigate the functional role of MRCCAT1 in ccRCC cells, we performed loss- or gain-of function experiments. First, three independent MRCCAT1-specific shRNAs were designed and transfected into786-O and Caki-1 cells to reduce MRCCAT1 expression (Fig. 3a). CCK-8 assay showed that MRCCAT1 knockdown inhibites the proliferation rate of 786-O and Caki-1 cells (Fig. 3b). Wound healing assay demonstrated that MRCCAT1 knockdown suppresses the migration ability of ccRCC cells compared with NC group (Fig. 3c and d). Moreover, Transwell invasion assays revealed that MRCCAT1 knockdown inhibites ccRCC cells invasion (Fig. 3e and f).

**Fig. 4:**
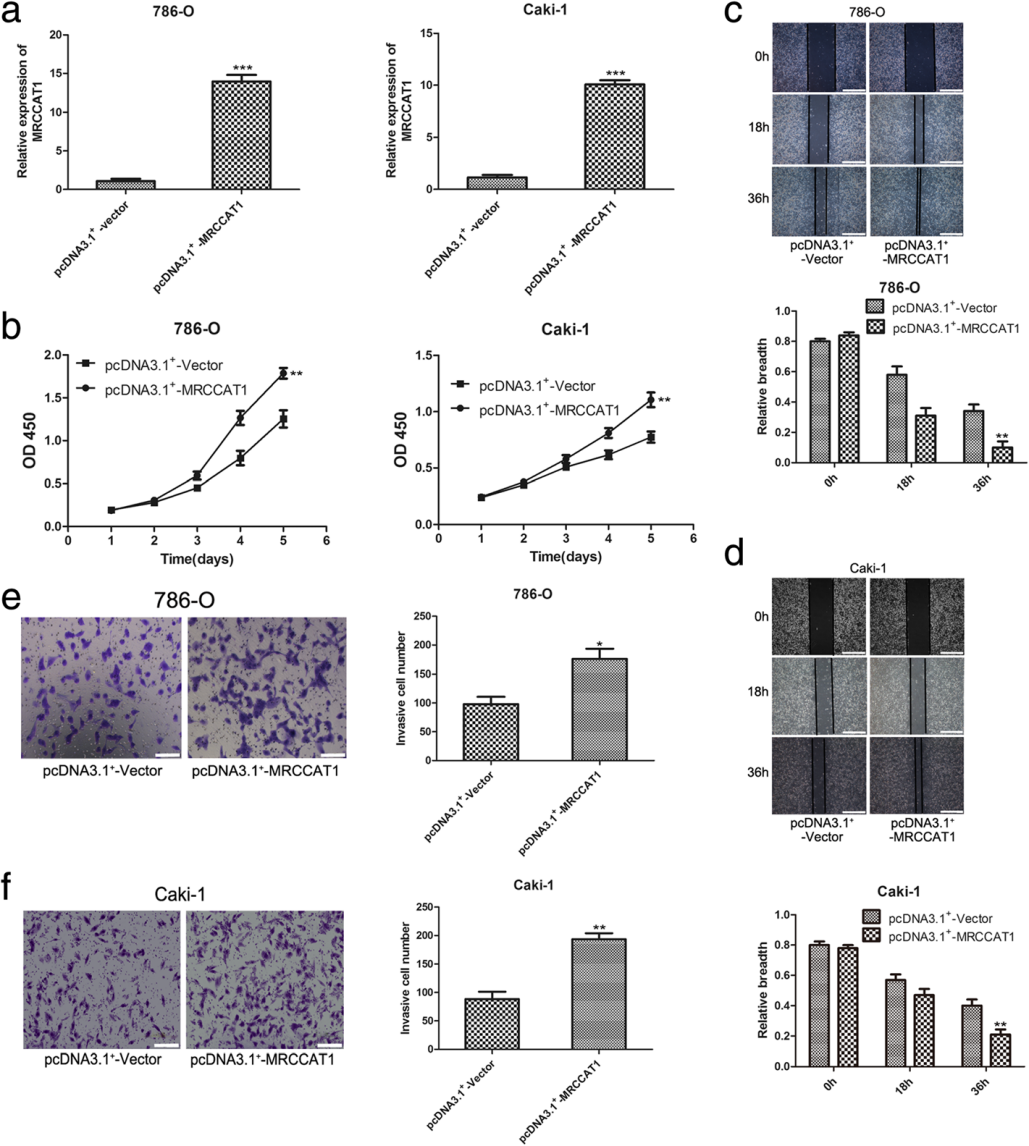
MRCCAT1 overexpression promotes ccRCC cells proliferation, migration and invasion in vitro. **a** Relative expression of MRCCAT1 in pcDNA3.1^+^-MRCCAT1 transfected compared with pcDNA3.1^+^-vector transfected 786-O and Caki-1 cells. **b** CCK-8 assay of MRCCAT1 overexpressed and NC ccRCC cells at indicated times. **c**, **d** Upper: representative images of the wound-healing assay of MRCCAT1 overexpressed compared with negative control 786-O (**c**) and Caki-1 cells (**d**), photographed at indicated times after scratching. Scale bar = 800 μm. Lower: the relative breath after migration was measured and calculated among the groups. **e**, **f** Left: Transwell assays were performed to evaluate the invasion of MRCCAT1 overexpressed compared with NC 786-O (**e**) and Caki-1 cells (**f**). Scale bar = 200 μm. Right: The statistical graph indicates the number of cells averaged from 8 random high power fields. The results are presented as the mean ± SD from three independent experiments; *P < 0.05, **P < 0.01, ***P < 0.001 by Student’s t-test

In parallel, the roles of MRCCAT1 overexpression in 786-O and Caki-1 cells were also investigated. Ectopic overexpression of MRCCAT1 was achieved by transfection of pcDNA3.1^+^-MRCCAT1 into 786-O and Caki-1 cells (Fig. 4a). CCK8 assay suggested that cell proliferation is enhanced by MRCCAT1 overexpression in 786-O and Caki-1 cells (Fig. 4b). Furthermore, MRCCAT1 overexpression promotes the migration and invasion of ccRCC cells (Fig. 4c-f). Together, these data indicated that MRCCAT1 could increase metastatic potential of ccRCC cells.

### Knockdown of MRCCAT1 suppresses the metastasis of ccRCC cells in vivo

**Fig. 5:**
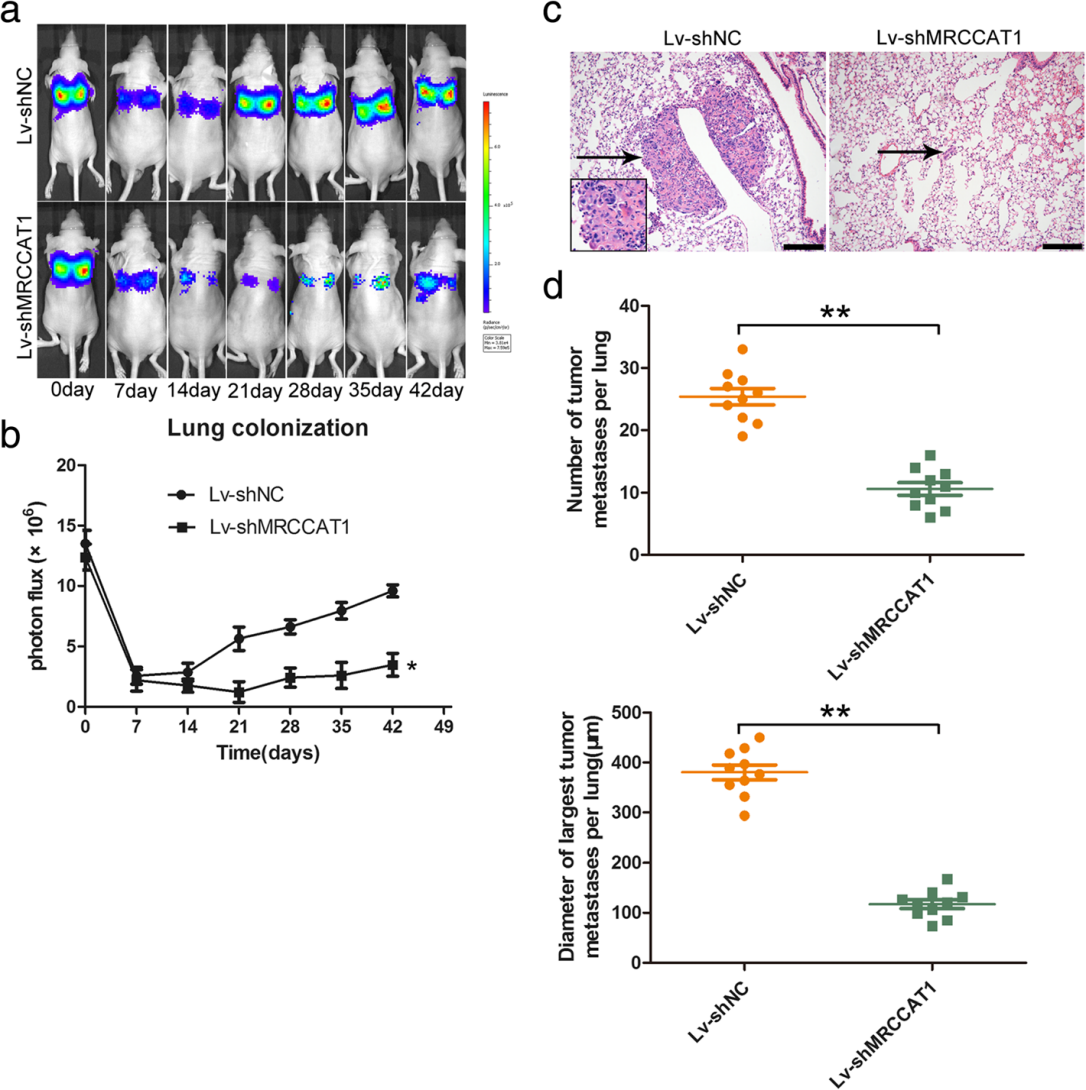
Knockdown of MRCCAT1 suppresses the metastasis of ccRCC cells in vivo. **a**, **b** Representative bioluminescent images (**a**) and quantification of bioluminescent imaging signal intensities (**b**) at each week after tail vein injection of MRCCAT1 knockdown and NC 786-O cells for 6 weeks. **c** Representative images of HE staining of metastatic nodules in the lungs of nude mice. The metastatic nodules are indicated by black arrows; Scale bar = 200 μm. **d** Upper: the numbers of metastatic tumor in nude mice lungs were calculated and compared. Lower: diameter of the largest metastatic tumor in nude mice lungs were calculated and compared. The results are presented as the mean ± SD for each group (*n* = 10). *P < 0.05, **P < 0.01 by Mann-Whitney U test

To examine the effect of MRCCAT1 on ccRCC metastasis in vivo, we established a lung metastatic mice model using MRCCAT1 stably depleted 786-O cells with luciferase expression by tail vein injection [26]. Compared with control group, bioluminescent signals were lower in the MRCCAT1 knockdown group (Fig. 5a and b). Six weeks later, less and smaller metastatic lesions were microscopically detected in the lungs of nude mice inoculated with MRCCAT1 knockdown cells compared with those inoculated with control cells (Fig. 5c and d). These results suggested that MRCCAT1 could promote pulmonary metastasis of ccRCC cells in vivo.

### MRCCAT1 promotes ccRCC cell metastasis by downregulating NPR3 expression

**Fig. 6:**
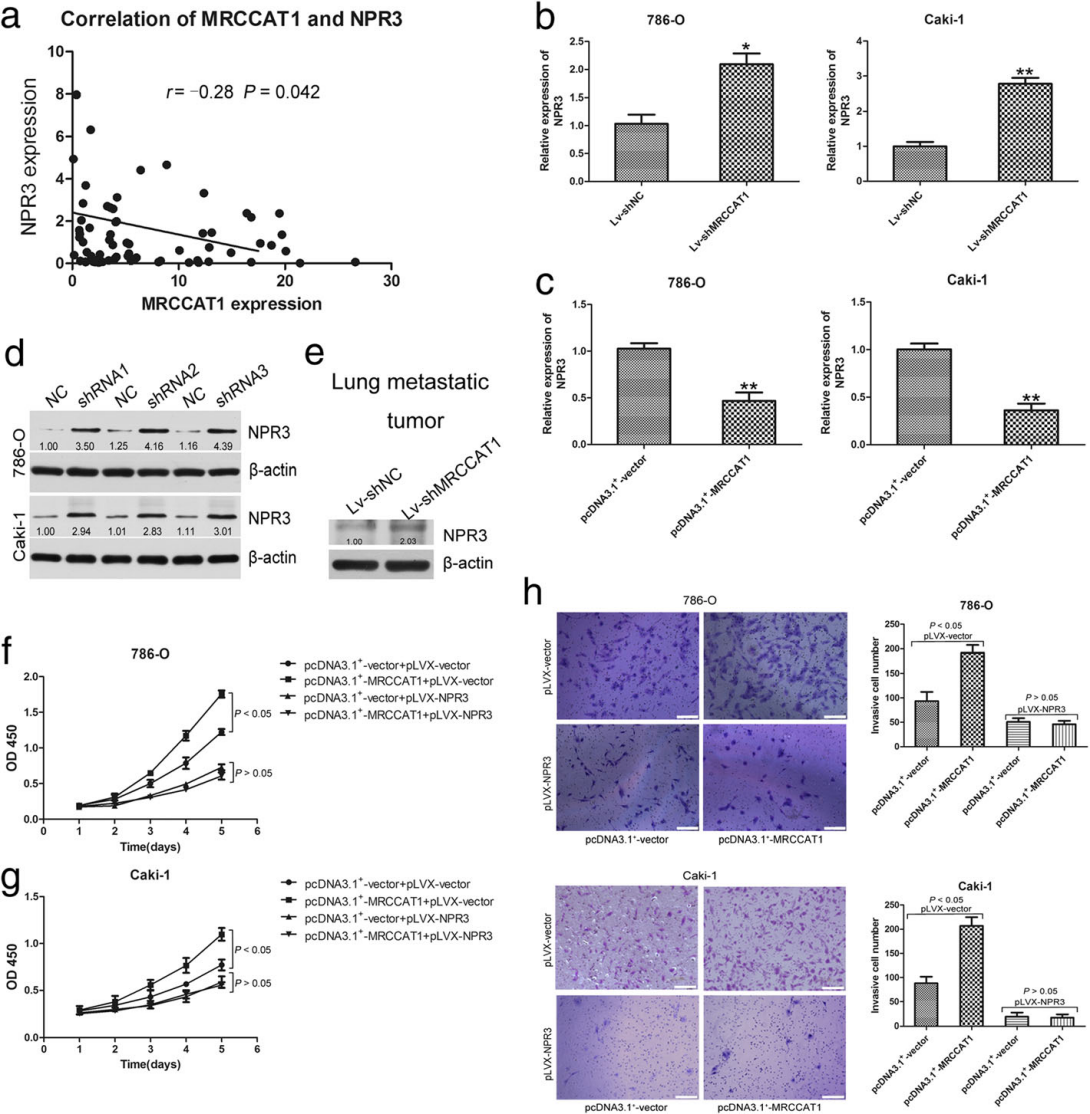
MRCCAT1 promotes ccRCC cell metastasis by downregulating NPR3 expression. **a** Correlation of MRCCAT1 and NPR3 at RNA levels in 68 ccRCC tumor samples. *r* = −0.28, *P* = 0.042 by Pearson correlation analysis. **b** Relative expression of NPR3 at mRNA levels between Lv-shNC and Lv-shMRCCAT1 cell lines. **c** Relative expression of NPR3 at mRNA levels between negative control and MRCCAT1 overexpression cell lines. **d** Western blot analysis of NPR3 upon MRCCAT1 knockdown. β-actin was used as an internal control. **e** Western blot analysis of NPR3 upon MRCCAT1 knockdown in lung metastatic tumor of nude mice. β-actin was used as an internal control. **f** CCK-8 assay in MRCCAT1 overexpressed and control 786-O cells transfected with pLVX-NPR3 at indicated times. **g** CCK-8 assay in MRCCAT1 overexpressed and control Caki-1 cells transfected with pLVX -NPR3 at indicated times. **h** Left: Transwell assays in MRCCAT1 overexpressed and control 786-O cells transfected with pLVX -NPR3. Scale bar = 200 μm. Right: The statistical graph indicates the number of cells from 8 random high power fields counted from three independent experiments. **i** Left: Transwell assays in MRCCAT1 overexpressed and control Caki-1 cells transfected with pLVX -NPR3. Scale bar = 100 μm. Right: The statistical graph indicates the number of cells averaged from 8 random high power fields. The results are presented as the mean ± SD from three independent experiments; *P < 0.05, **P < 0.01 by Student’s t-test

An lncRNAs-mRNAs co-expression network was built according to the normalized signal intensity of specific expression genes in the microarray. For each pair of genes, we calculated the Pearson correlation and chose the significant correlated pairs to construct the network (Additional file 3: Figure S2). In this lncRNAs-mRNAs co-expression network, we found that MRCCAT1 is negatively correlated with NPR3. To verify the prediction, we examined the RNA level of MRCCAT1 and NPR3 in 68 clinical ccRCC samples. Real-time PCR data showed that the expression of MRCCAT1 is negatively correlated with NPR3 (*P* = 0.042, *r* = −0.28), in line with the lncRNAs-mRNAs co-expression network (Fig. 6a).

Next, we examined whether MRCCAT1 could negatively regulate NPR3 expression in ccRCC cells. As shown in Fig. 6b, Real-time PCR data showed that NPR3 mRNA level is significantly increased in MRCCAT1 knockdown 786-O and Caki-1 cells. While, the NPR3 mRNA level is significantly decreased upon MRCCAT1 overexpression in 786-O and Caki-1 (Fig. 6c). Furthermore, western blot analysis revealed that the protein level of NPR3 is markedly increased in response to MRCCAT1 knockdown in 786-O and Caki-1 cells (Fig. 6d). Additionally, NPR3 expression is elevated in MRCCAT1 knockdown lung metastatic tumors (Fig. 6e). These data suggest that the NPR3 expression is inhibited by MRCCAT1 in ccRCC cells.

Based on these observations, we further sought to determine whether NPR3 was responsible for the pro-metastatic role of MRCCAT1. As shown in Fig. 6f and g, induction of NPR3 could abolish the role of MRCCAT1 in promoting cell proliferation. Consistently, the enhanced cell invasion by overexpression of MRCCAT1 is reversed by coexpression of NPR3 (Fig. 6h and i). Together, these data indicated that MRCCAT1 promotes ccRCC progression by suppressing NPR3.

### MRCCAT1 suppresses NPR3 to potentiate p38-MAPK signaling

**Fig. 7:**
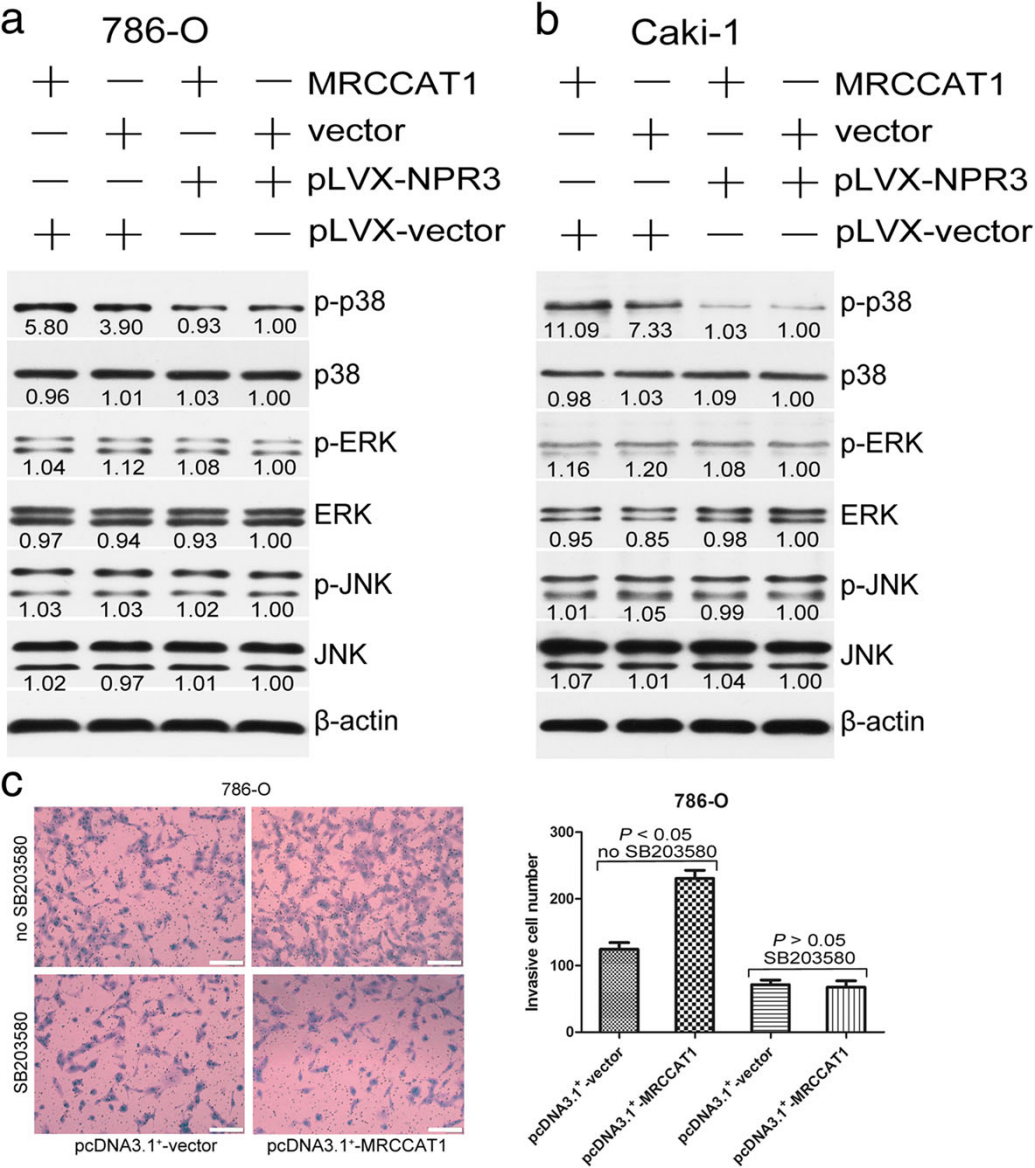
MRCCAT1 suppresses NPR3 to potentiate p38- MAPK signaling. **a** Western blot analysis of indicated proteins in MRCCAT1 overexpressed and control 786-O cells transfected with pLVX -NPR3. β-actin was used as an internal control. **b** Western blot analysis of indicated proteins in MRCCAT1 overexpressed and control Caki-1 cells transfected with pLVX -NPR3. β-actin was used as an internal control. **c** Left: Transwell assays in MRCCAT1 overexpressed and control 786-O cells treated with SB203580. Scale bar = 200 μm. Right: The statistical graph indicates the number of cells averaged from 8 random high power fields. The results are presented as the mean ± SD from three independent experiments. *P* values were calculated by Student’s t-test

NPR3 has previously been shown to play a pivotal role in tumor proliferation and metastasis by being negatively coupled to adenylyl cyclase and MAPK signaling pathways [11–13]. Adenylyl cyclase can result in stimulation of mitogen-activated protein kinase (MAPK) signal pathways [27, 28]. To address whether MAPK signaling pathways were involved in the function of MRCCAT1, the phosphorylation levels of ERK, p38 and JNK were detected by Western blot. We found that phosphorylated p38 is increased in MRCCAT1 overexpressed cells and could be reversed by NPR3 induction, while the phosphorylation levels of ERK and JNK are unchanged (Fig. 7a and b). Furthermore, transwell invasion assay revealed that MRCCAT1-enhanced cell invasion could be perturbed by SB203580 (50 μmol/L) treatment, a p38-MAPK inhibitor (Fig. 7c). Taken together, these data suggested that MRCCAT1 promotes ccRCC metastasis by negatively regulating NPR3 expression and then activating p38-MAPK signaling pathway.

### MRCCAT1 suppresses NPR3 expression by recruiting polycomb repressive complex 2 to *NPR3* promoter

**Fig. 8:**
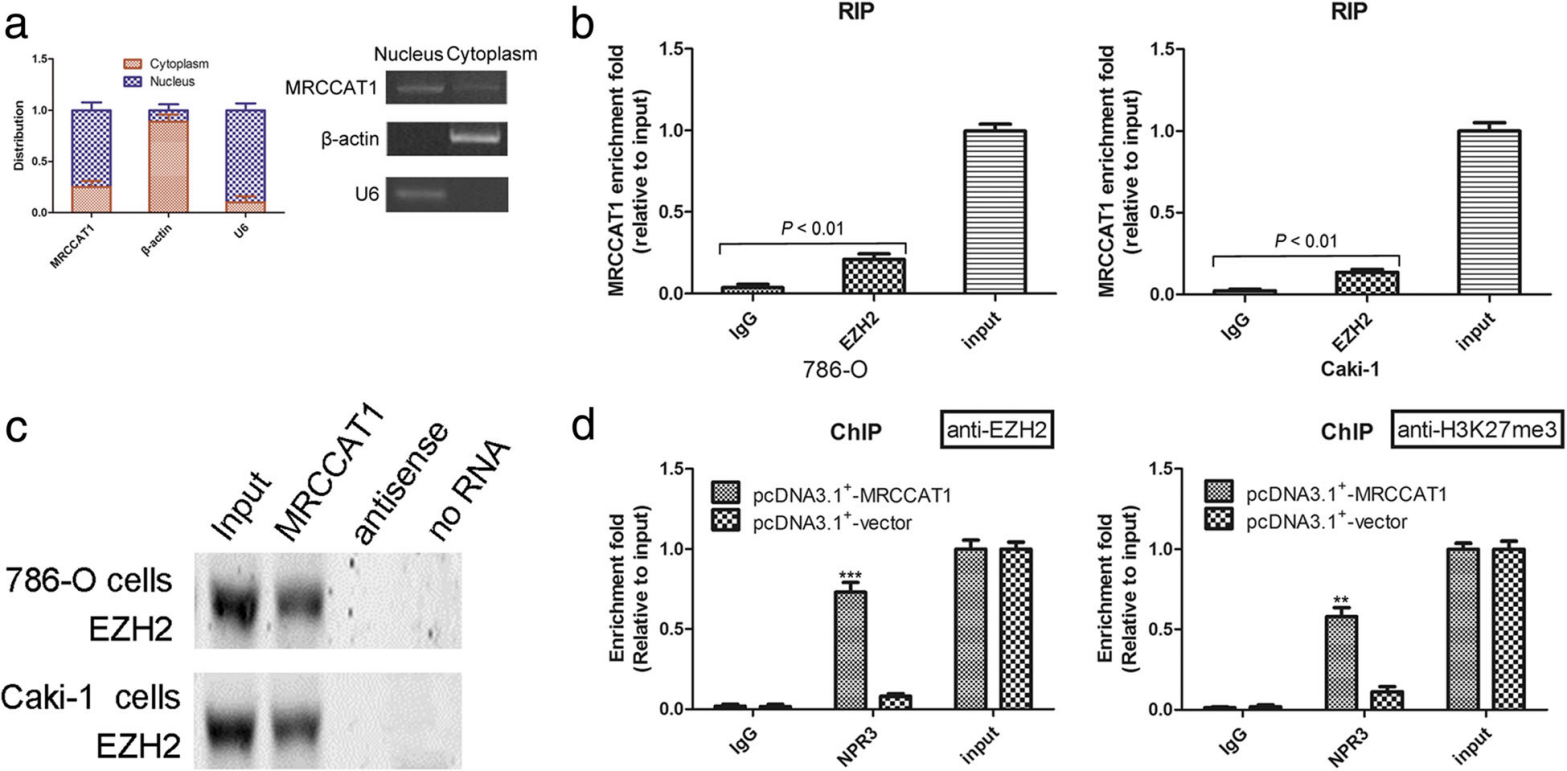
MRCCAT1 suppresses NPR3 expression by recruiting Polycomb Repressive Complex 2 to *NPR3* promoter. **a** qRT-PCR analysis of MRCCAT1 in subcellular fraction of Caki-1 cells. U6 and β-actin acted as nucleus and cytoplasm marker, respectively. **b** RIP assay analysis of the enrichment of MRCCAT1 to EZH2 in 786-O and Caki-1 cells. **c** Biotinylated MRCCAT1 or antisense RNA were incubated with nuclear extracts of 786-O and Caki-1 cells, targeted with streptavidin beads, and washed. Then the associated proteins were resolved in a gel. Western blot analysis of the specific association of EZH2 and MRCCAT1. **d** ChIP assays were conducted on *NPR3* promoter regions using the indicated antibodies. Enrichment was determined relative to input controls. The results are presented as the mean ± SD from three independent experiments; **P < 0.01, ***P < 0.001 by Student’s t-test

To investigate the mechanisms by which MRCCAT1 regulate NPR3, we first examined the subcellular localization of MRCCAT1. As shown in Fig. 8a, MRCCAT1 is predominately localized in nucleus. Recent studies have reported that lncRNAs in nucleus could recruit polycomb-group proteins to regulate gene expression [7, 17]. Twenty percent of all human lncRNAs have been shown to physically associate with Polycomb Repressive Complex 2 (PRC2) [29], which comprises of SUZ12, EED, EZH1/2 (H3K27 methyltransferase) and RbAp16/48, and represses gene transcription by inducing trimethylation of H3K27. Some lncRNAs have been shown to act in *trans* to alter the target specificity of PRC2 and thus inhibit a number of anti-metastatic genes [7]. Thus, we hypothesized that MRCCAT1 might repress *NPR3* expression in such manner. To test this, we performed RIP assay with an antibody against EZH2 (an important subunit of the PRC2 complex), and found a significant enrichment of MRCCAT1 with EZH2, compared with IgG control (Fig. 8b). Moreover, RNA pull-down further confirmed the interaction between MRCCAT1 and EZH2 (Fig. 8c). To further address whether MRCCAT1 repressed *NPR3* transcription through recruiting EZH2 to *NPR3* promoters, we conducted ChIP analysis in MRCCAT1-overexpressing 786-O cells. ChIP assay demonstrated that MRCCAT1 increases the binding of EZH2 and H3K27me3 at *NPR3* promoter regions (Fig. 8d), indicating that MRCCAT1 bound to EZH2 to repress *NPR3* transcription.

## Discussion

Metastatic ccRCC patients have poor prognosis and limited clinical therapeutic options at present. Hence, it is necessary to investigate the biological basis of metastatic ccRCC and identify novel targets for metastasis prevention and therapy. In this study, we identified a novel lncRNA MRCCAT1 which is highly expressed in metastatic ccRCC. Our data showed that MRCCAT1 promoted ccRCC metastasis by inhibiting *NPR3* transcription and activating p38-MAPK signaling.

lncRNAs have been shown to play an important role in diverse biologic processes such as development, cell growth, and tumorigenesis [30]. More recently, lncRNAs have also been implicated in regulating specific steps in the metastatic cascade [7, 31]. Metastatic ccRCC is a significant challenge for the clinical management of RCC. The molecular mechanisms underlying metastatic spread of ccRCC are unclear, limiting the development of effective pharmacological therapies for advanced ccRCC. In the present study, we found a new lncRNA transcript, MRCCAT1, which was significantly upregulated in metastatic ccRCC tissues through the lncRNA expression microarray. The survival analysis revealed that MRCCAT1 was correlated with shorter survival time of ccRCC patients. Additionally, multivariate analysis showed that high expression of MRCCAT1, tumor size and metastases were each independent risk factors for overall patient survival rate following surgery. These results suggested that MRCCAT1 may be a novel risk biomarker for judging ccRCC prognosis. Furthermore, our functional experiments demonstrated that MRCCAT1 had an important role in metastasis of ccRCC cells. MRCCAT1 knockdown could suppress ccRCC cell invasion in vitro and metastasis in vivo. In general, these data indicated that MRCCAT1 may be closely related to metastasis and act as a metastasis enhancer in ccRCC.

Metastasis is a crucial factor in the determination of prognosis and survival of ccRCC patients. Thus, elucidating the molecular mechanisms underlying ccRCC metastasis is urgently needed. We found that MRCCAT1 expression was negatively associated with NPR3 level in an lncRNAs-mRNAs co-expression network. Further study found that MRCCAT1 overexpression could decrease the mRNA levels of NPR3. In the experiments of co-transfection of MRCCAT1 and NPR3, the results revealed that MRCCAT1 produced a marked effect of promoting ccRCC metastasis, which was depending on inhibiting NPR3 expression. Recent studies suggest that NPR3 may be negatively coupled to adenylyl cyclase and MAP kinase signaling pathway (mitogen-activated protein kinase, MAPK) [11, 12]. Adenylyl cyclase can result in stimulation of MAPK signal pathways [27, 28]. Several studies also show the activation of MAPK signaling pathway in tumorigenesis, metastasis and angiogenesis of multiple human malignancies, including renal cell carcinoma (RCC) [32–34].

Our results also indicated that MRCCAT1 inhibits the mRNA levels of NPR3 through binding to EZH2, and then promotes the activation of p38-MAPK signaling and ccRCC cell metastasis. Recent studies also suggest that some lncRNAs act *in trans* to alter the target specificity of PRC2 and thus repress a number of anti-metastatic genes [7]. PRC2 is comprised of SUZ12, EED, EZH1/2 (H3K27 methyltransferase) and RbAp16/48, and represses gene transcription by inducing trimethylation of H3K27. The SET domain present in EZH2 is responsible for methylation of Lys 27 of histone H3 [35, 36].

Taken together, the results from this study suggest that MRCCAT1 orchestrate intricate adenylyl cyclase and p38-MAPK signaling pathways by negatively regulating NPR3 to modulate metastasis in ccRCC. Thus, MRCCAT1 may serve as a potential treatment target in the metastatic ccRCC. There are some limitations of our study, including the limited size of cohort and limited functional studies of MRCCAT1 in vivo and in vitro. The underlying mechanism for MRCCAT1 up-regulation in metastatic ccRCC also remain unclear and should be elucidated in the future.

## Conclusions

In summary, MRCCAT1 plays a critical role in promoting cell metastasis of ccRCC by negatively regulating NPR3 expression and activating the p38-MAPK signaling pathway. MRCCAT1 could serve as an independent predictor for clinical outcomes in ccRCC patients. Based on these findings, MRCCAT1 may represent a potential therapeutic target to curb the progression of ccRCC.

## Additional files

Additional file 1: Table S1. Sequences of primers used for plasmid construction in this study. **Table S2.** Sequences of primers used for qRT-PCR in this study. (DOCX 19 kb)

Additional file 2: Figure S1. Full-length of MRCCAT1 and prediction of protein-coding potential. a The nucleotide sequence of full-length human MRCCAT1 was 1718 bp. b Putative proteins possibly encoded by MRCCAT1 were predicted by the ORF Finder. c The codon substitution frequency scores (PyhloCSF) of MRCCAT1. (TIFF 8749 kb)

Additional file 3: Figure S2. Gene chip co-expression network. LncRNAs-mRNAs co-expression network was constructed according to the normalized signal intensity of specific expression genes. Dots represent genes, rounded rectangles represent lncRNAs (red, up-regulation; blue, down-regulation), and lines represent the regulatory relationship between them (solid lines represent positive regulation, the dotted lines represent negative regulation). (TIFF 3478 kb)

## Abbreviations

CCK-8: Cell counting Kit-8
ccRCC: Clear cell renal cell carcinoma
EZH2: Enhancer of zeste homolog 2
MAP: Mitogen-activated protein
MAPK: Mitogen-activated protein kinase
PRC2: Polycomb repressive complex 2
qRT-PCR: Quantitative real-time PCR
RIP: RNA immunoprecipitation
shRNA: Short hairpin RNA
