# Supplementary material for: Long noncoding RNA MRCCAT1 promotes metastasis of clear cell renal cell carcinoma via inhibiting NPR3 and activating p38-MAPK signaling
Source: Mol Cancer. 2017 Jun 28;16:111. doi: 10.1186/s12943-017-0681-0 (PMC5490088; doi:10.1186/s12943-017-0681-0)
Supplement: Supplementary file 1 — Sequences of primers used for plasmid construction in this study. Table S2. Sequences of primers used for qRT-PCR in this study. (DOCX 19 kb) [file 12943_2017_681_MOESM1_ESM.docx]

**Additional file**

| **Table S1** Sequences of primers used for plasmid construction in this study | | | |
| --- | --- | --- | --- |
| MRCCAT1 shRNA1 | Sense (5’-3’) | GatccGCCACTACACAGCACTGCTTTTCAAGAGAAAGCAGTGCTGTGTAGTGGTTTTTTc | |
|  | Antisense (5’-3’) | aattgAAAAAACCACTACACAGCACTGCTTTCTCTTGAAAAGCAGTGCTGTGTAGTGGCg | |
| MRCCAT1  shRNA2 | Sense (5’-3’) | GatccGCTTCCAGCCCAGAACTTTTTCAAGAGAAAAGTTCTGGGCTGGAAGCTTTTTTc | |
|  | Antisense (5’-3’) | aattgAAAAAAGCTTCCAGCCCAGAACTTTTCTCTTGAAAAAGTTCTGGGCTGGAAGCg | |
| MRCCAT1 shRNA3 | Sense (5’-3’) | GatccGCCCAAAGCCATCTGGTTATTCAAGAGATAACCAGATGGCTTTGGGCTTTTTTc | |
|  | Antisense (5’-3’) | aattgAAAAAAGCCCAAAGCCATCTGGTTATCTCTTGAATAACCAGATGGCTTTGGGCg | |
| Control shRNA | Sense (5’-3’) | gatccTTCTCCGAACGTGTCACGTAATTCAAGAGATTACGTGACACGTTCGGAGAATTTTTTg | |
|  | Antisense (5’-3’) | aattcAAAAAATTCTCCGAACGTGTCACGTAATCTCTTGAATTACGTGACACGTTCGGAGAAg | |
| pcDNA3.1^+^- MRCCAT1 | Sense (5’-3’) | aaGGTACCTAGCCATCTCCGTTTTCAAAT | |
|  | Antisense (5’-3’) | aaCTCGAGAGAGAGAGAGGGAAGACACTGT | |
| pLVX-NPR3 | Sense (5’-3’) | CCGGAATTCGCCACCATGCCGTCTCTGCTGGTGC | |
|  | Antisense (5’-3’) | CCGGGATCCAGCTACTGAAAAATGGGATCTGATGGA | |

| **Table S2** Sequences of primers used for qRT-PCR in this study | | |
| --- | --- | --- |
| MRCCAT1 | Forward (5’-3’) | CAGCCGTTCAGAATCTCCTGT |
|  | Reverse (5’-3’) | AACCAGATGGCTTTGGGCAG |
| ATF3_003 | Forward (5’-3’) | CTTCAGTATTAGCAGAGCCACAGG |
|  | Reverse (5’-3’) | TCTCCGACTCCTGGGACACA |
| TPM4_006 | Forward (5’-3’) | AGGCCTGAGCTTCTCAAACT |
|  | Reverse (5’-3’) | AAAAATGTCCCCTACTTTCCCT |
| ELL2_004 | Forward (5’-3’) | TCCCTCCGACTTCTTTCCCT |
|  | Reverse (5’-3’) | GCAACATTCAGAGCCGACTG |
| NPR3 | Forward (5’-3’) | CTCCCTGCAAATCATGTGGC |
|  | Reverse (5’-3’) | GGGTTCGCCTCTCAATGGTT |
| β-actin | Forward (5’-3’) | GTGGACATCCGCAAAGAC |
|  | Reverse (5’-3’) | AAAGGGTGTAACGCAACTA |
| U6 | Forward (5’-3’) | CTCGCTTTGGCAGCACA |
|  | Reverse (5’-3’) | AACGCTTCACGAATTTGCGT |
