# Supplementary figures and images for: Long noncoding RNA MRCCAT1 promotes metastasis of clear cell renal cell carcinoma via inhibiting NPR3 and activating p38-MAPK signaling
Source: Mol Cancer. 2017 Jun 28;16:111. doi: 10.1186/s12943-017-0681-0 (PMC5490088; doi:10.1186/s12943-017-0681-0)

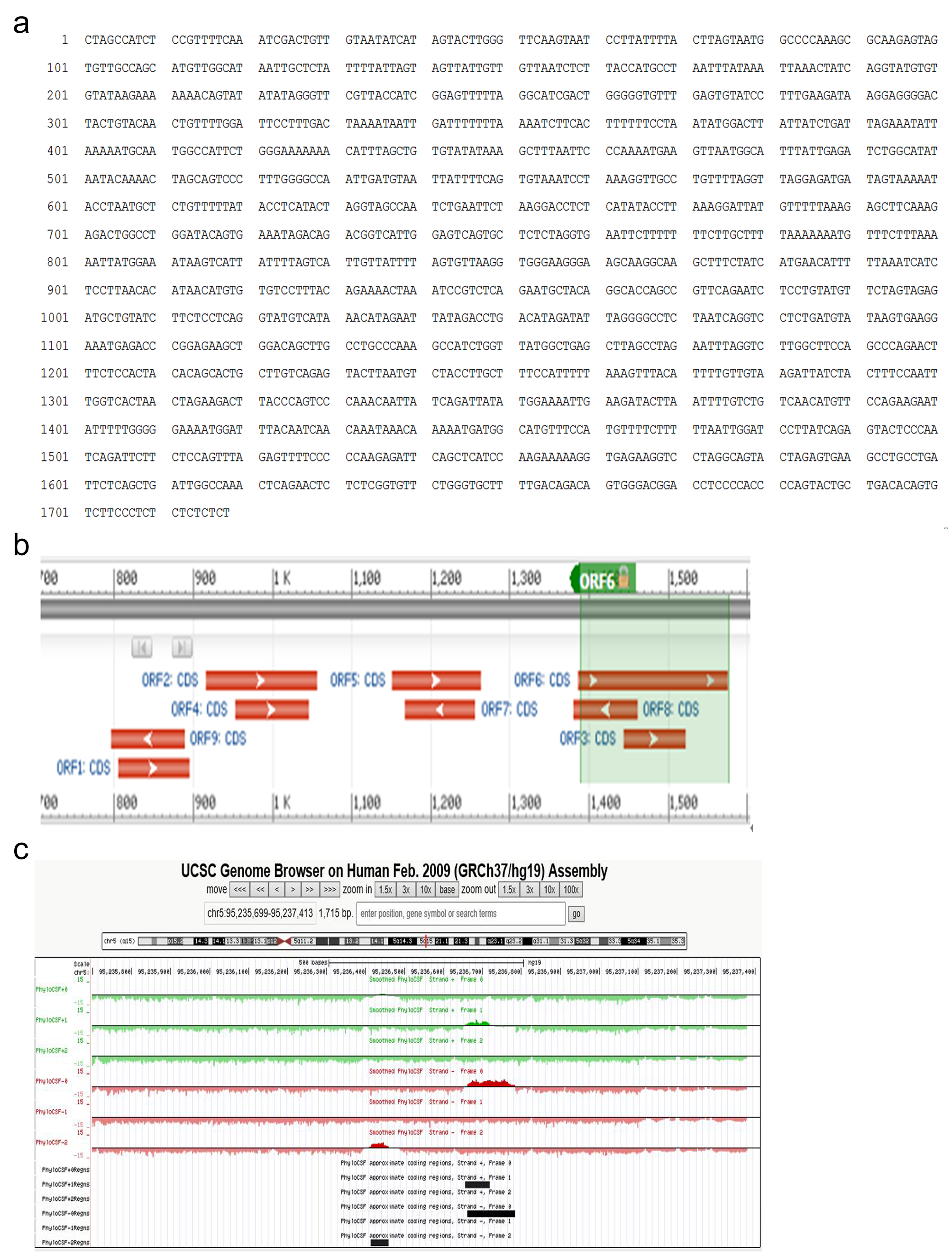

Supplement: Supplementary file 2 — Full-length of MRCCAT1 and prediction of protein-coding potential. a The nucleotide sequence of full-length human MRCCAT1 was 1718 bp. b Putative proteins possibly encoded by MRCCAT1 were predicted by the ORF Finder. c The codon substitution frequency scores (PyhloCSF) of MRCCAT1. (TIFF 8749 kb) [file 12943_2017_681_MOESM2_ESM.tif]

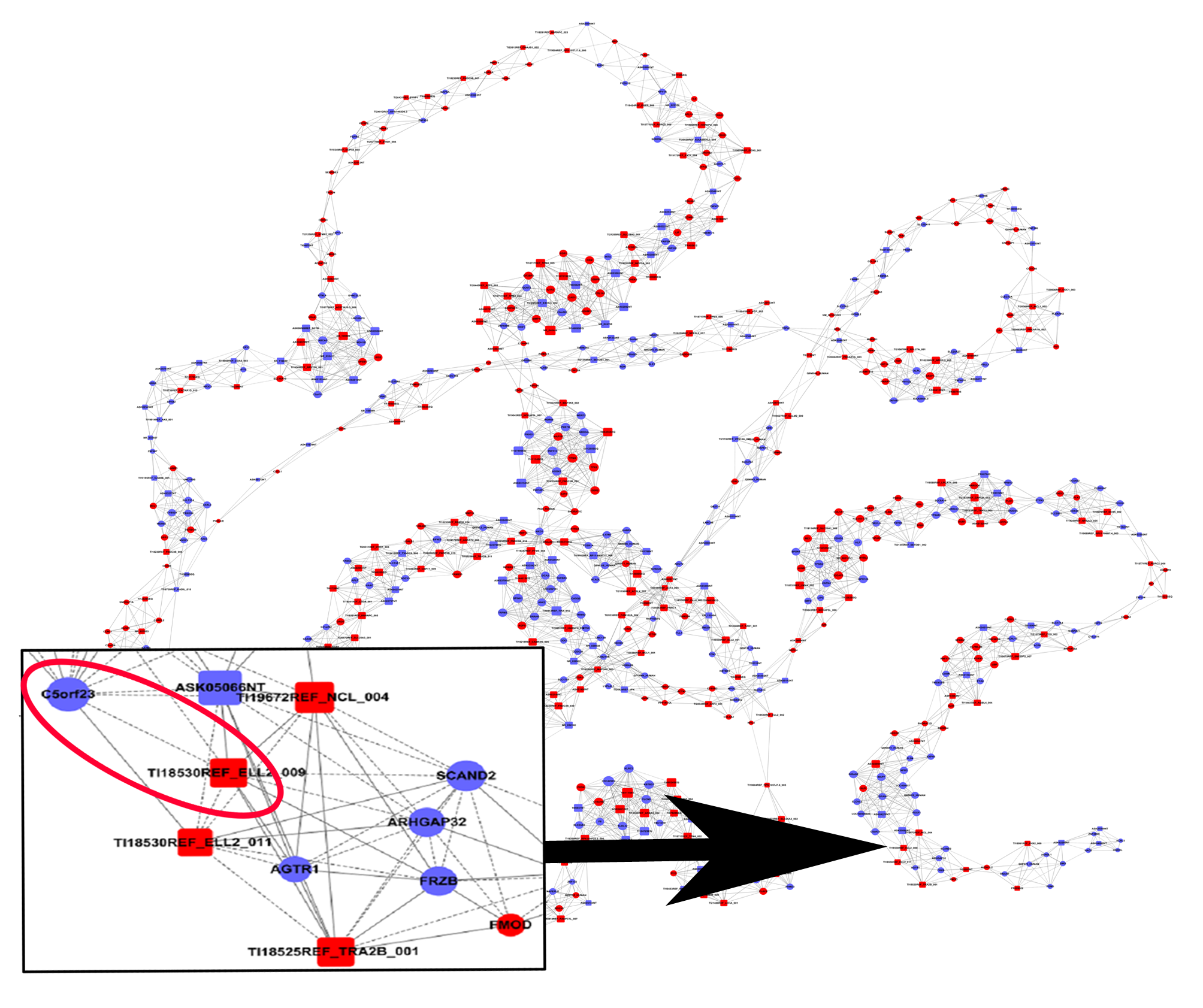

Supplement: Supplementary file 3 — Gene chip co-expression network. LncRNAs-mRNAs co-expression network was constructed according to the normalized signal intensity of specific expression genes. Dots represent genes, rounded rectangles represent lncRNAs (red, up-regulation; blue, down-regulation), and lines represent the regulatory relationship between them (solid lines represent positive regulation, the dotted lines represent negative regulation). (TIFF 3478 kb) [file 12943_2017_681_MOESM3_ESM.tif]
